# Mixed Signals: Co-Stimulation in Invariant Natural Killer T Cell-Mediated Cancer Immunotherapy

**DOI:** 10.3389/fimmu.2017.01447

**Published:** 2017-11-01

**Authors:** Susannah C. Shissler, Michael S. Lee, Tonya J. Webb

**Affiliations:** ^1^Department of Microbiology and Immunology, Greenebaum Comprehensive Cancer Center, University of Maryland School of Medicine, Baltimore, MD, United States

**Keywords:** invariant natural killer T, co-stimulation, cancer, immunotherapy, chimeric antigen receptor, checkpoint, natural killer T

## Abstract

Invariant natural killer T (iNKT) cells are an integral component of the immune system and play an important role in antitumor immunity. Upon activation, iNKT cells can directly kill malignant cells as well as rapidly produce cytokines that stimulate other immune cells, making them a front line defense against tumorigenesis. Unfortunately, iNKT cell number and activity are reduced in multiple cancer types. This anergy is often associated with upregulation of co-inhibitory markers such as programmed death-1. Similar to conventional T cells, iNKT cells are influenced by the conditions of their activation. Conventional T cells receive signals through the following three types of receptors: (1) T cell receptor (TCR), (2) co-stimulation molecules, and (3) cytokine receptors. Unlike conventional T cells, which recognize peptide antigen presented by MHC class I or II, the TCRs of iNKT cells recognize lipid antigen in the context of the antigen presentation molecule CD1d (Signal 1). Co-stimulatory molecules can positively and negatively influence iNKT cell activation and function and skew the immune response (Signal 2). This study will review the background of iNKT cells and their co-stimulatory requirements for general function and in antitumor immunity. We will explore the impact of monoclonal antibody administration for both blocking inhibitory pathways and engaging stimulatory pathways on iNKT cell-mediated antitumor immunity. This review will highlight the incorporation of co-stimulatory molecules in antitumor dendritic cell vaccine strategies. The use of co-stimulatory intracellular signaling domains in chimeric antigen receptor-iNKT therapy will be assessed. Finally, we will explore the influence of innate-like receptors and modification of immunosuppressive cytokines (Signal 3) on cancer immunotherapy.

## Invariant Natural Killer T (iNKT) Cells and Co-Stimulation

Natural killer T (NKT) cells exhibit similar traits to their namesakes. They express cell surface markers similar to natural killer (NK) cells such as CD161, CD56, and CD16. As a subset of T cells, NKT cells develop in the thymus and possess a T cell receptor (TCR) (1). Unlike conventional T cells, the NKT TCR recognizes lipid antigens in the context of the MHC class Ib molecule, CD1d (2). CD1d is expressed on many types of epithelial and endothelial cells and antigen-presenting cells (APCs), such as B cells and dendritic cells (DCs) (3, 4). There are two subsets of NKT cells that are differentiated by their TCRs. Type I NKT (iNKT) cells have an invariant TCR whereas type II have diverse TCRs (5–8). The TCR of iNKT cells is composed of a single α chain (Vα14Jα18 in mice and Vα24Jα18 in humans) paired with β chains of limited diversity (Vβ8.2, 7 or 2 in mice and Vβ11 in humans) (9). While endogenous activating and suppressive antigens remain contested, iNKT cells respond to the exogenous glycolipid antigen, α-galactosylceramide (α-GalCer), while type II NKT cells do not (10). Upon antigenic stimulation, iNKT cells once again mirror NK and T cells. As innate-like lymphocytes, they respond to antigenic stimulation within a few hours by producing large amounts of Th1, Th2, and Th17 cytokines (11). This rapid response can be attributed to their storage of cytokine mRNA before activation (12). Like both NK cells and cytotoxic T lymphocytes (CTLs), iNKT cells can be directly cytotoxic (13). This combination of effector functions allows them to address stimuli directly and incite the immune system at large to mount an effective immune response against various assaults.

**Figure 1:**
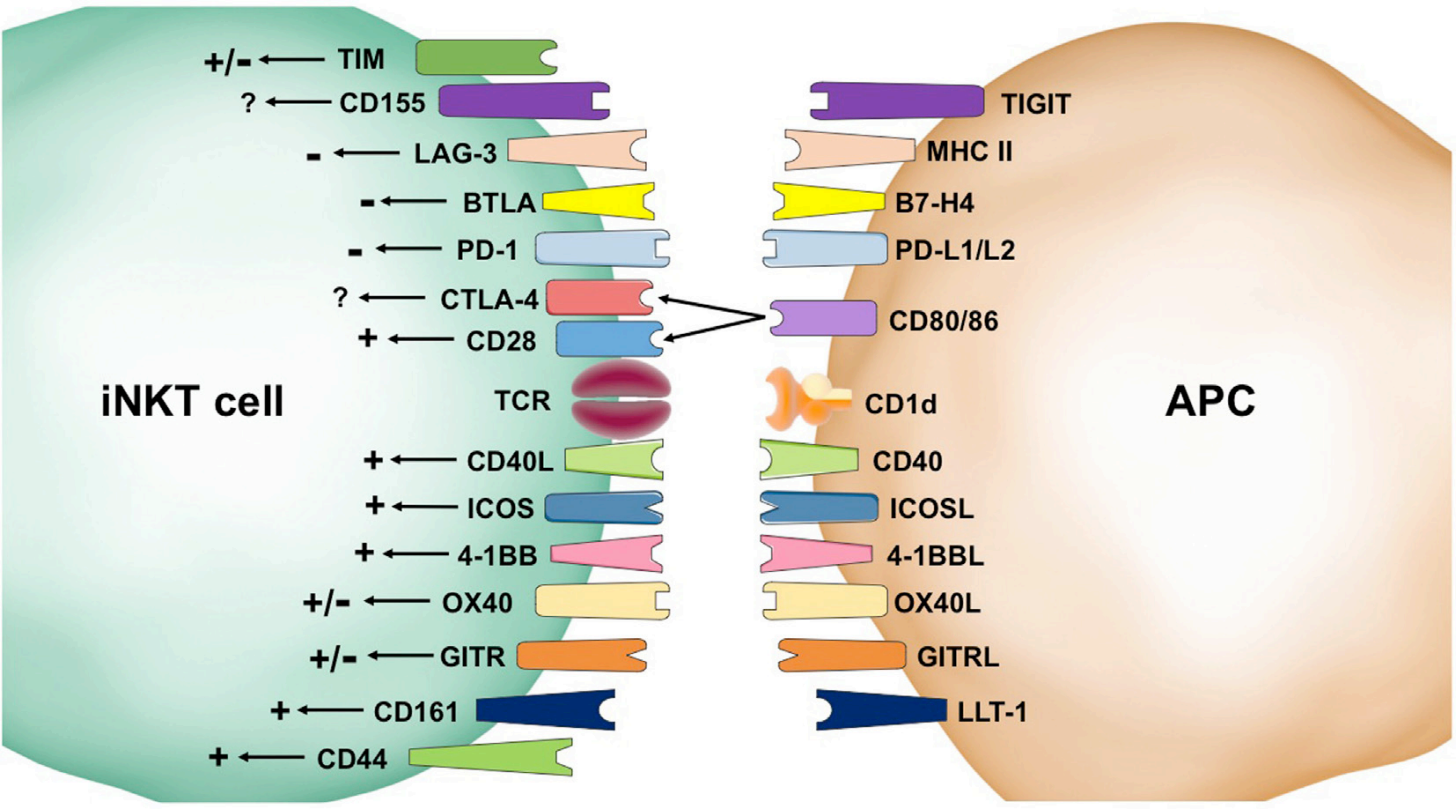
Second signals provided by co-receptors influence invariant natural killer T (iNKT) cell biology. Stimulatory (+) pathways result in homeostatic survival and enhanced activation, cytokine production, expansion, and cytotoxicity. These positive signals come from CD44, CD161, OX40, 4-1BB, ICOS, CD40L, and CD28. Inhibitory (−) signaling can result in cell death and inhibition of iNKT cell activation. Receptors that have shown negative signaling effects include programmed death (PD)-1, B and T lymphocyte attenuator (BTLA), and lymphocyte activation gene (LAG-3). The impacts of co-receptors, such as T cell immunoglobulin mucin (TIM), CD155, CTLA-4, OX40, and GITR, are not settled in the literature and are indicated by a +/− symbol. Some co-receptors, such as CD40L, selectively skew the immune response.

Naïve T cells conventionally require three signals for effective activation. Signal 1 is TCR: antigen–MHC engagement, Signal 2 is co-stimulatory molecules, and Signal 3 is cytokine stimulation. Signals 1 and 2 are regarded as mandatory for activation whereas Signal 3 is thought to direct the immune response (14, 15). This review focuses on the importance of Signal 2 for type I iNKT cell activation and function in antitumor immunity. Because the majority of the references presented herein refer to mouse iNKT cells, it will be explicitly stated when the data refer to human iNKT cells. Co-stimulation receptors can provide multiple types of signals, including positive/stimulatory and negative/inhibitory, and influence the type of response. There are two primary families of co-receptors: the CD28/B7 family and the TNF receptor superfamily (TNFRSF). The CD28/B7 family members are composed of immunoglobulin domains whereas the TNFRSF members have cysteine-rich extracellular domains (15). First, we will review the literature that addresses the effects of co-stimulatory receptors on iNKT cell biology, which are summarized in Figure 1.

CD28 is the canonical co-stimulatory molecule referred to during T cell activation. It is known to compete with CTLA-4, an inhibitory signaler, to interact with CD80/86 (also known as B7-1/2). In iNKT cells, CD28 is important for expansion during thymic development and after stimulation in the periphery (16–19). CD28 is constitutively expressed on iNKT cells, but its expression is downregulated during anergy or exhaustion (20). Blockade of CD80/86 suppresses production of both Th1 and Th2 cytokines and immune responses (21). In an experimental autoimmune encephalomyelitis model, blockade of CD86 during α-GalCer activation resulted in a Th2 bias (22). The literature available clearly shows a stimulatory role for CD28 signaling in iNKT cells.

Inducible T cell costimulator (ICOS or CD278), another member of the CD28 family, is constitutively expressed on iNKT cells, and its expression is increased after activation (23). The ICOS:ICOSL pathway is important for homeostatic proliferation and Th1 and Th2 immune responses (23, 24). During stimulation by marginal zone B cells, ICOS:ICOSL interactions are necessary to produce Th2 cytokines (25). While ICOS engagement is distinctly positive, its influence over the immune response is uncertain.

CD40L, a member of the TNFRSF that interacts with CD40 on APCs, has both positive co-stimulatory abilities as well as influence over the type of immune response generated. iNKT cells have been shown to provide cognate help to B cells that is independent of CD40:CD40L interactions (26) and non-cognate help in a method dependent on CD40:CD40L interactions (27). CD40 is upregulated after activation and necessary for production of a Th1 inflammatory response in intracellular infections (28) and antitumor immunity (29, 30). Th2 cytokine production decreases in NKT cells activated by APCs that had been treated with an agonistic anti-CD40 antibody (22). Blockade of CD40L results in decreased Th1 responses (21) and increased Th2 responses (31) making this pathway a likely target for enhancing transplant tolerance. CD40L signaling is distinctly positive and demonstrates importance for Th1 immune responses.

4-1BB (CD137), another member of the TNFRSF, is expressed on iNKT cells after activation. 4-1BB stimulation during or after activation results in increased cytokine production (32) and enhanced iNKT cell proliferation in mice and humans (33). Under resting conditions, 4-1BB:4-1BBL interactions between iNKT cells and monocytes in the lungs provide homeostatic survival signals for both cell types in both human and mouse models (33). Blockade of 4-1BB results in decreased immune responses, including Th1 and Th2 responses (34). 4-1BB signaling is an important stimulatory pathway for iNKT cell function.

Although CD44 is expressed on all T cells, its function differs in iNKT cells. Unlike in conventional T cells, iNKT cell CD44 can bind hyaluronic acid and induce activation. Crosslinking CD44 results in iNKT cell activation and increased cytokine production as well as protection from activation-induced cell death (35). Stimulation of iNKT cells with artificial antigen-presenting cells that only possess CD1d and anti-CD44 on their surface results in potent iNKT cell cytokine production (36). Human iNKT cells also express CD161, which is a C-type lectin receptor that interacts with lectin-like transcript-1. While CD161 crosslinking by itself does not induce activation, CD161 blockade decreases cytokine production and proliferation. iNKT cell mediated cytotoxicity is independent of CD161 (37). CD44 and CD161 exert a positive influence over iNKT cell activation.

Glucocorticoid-induced TNFR-related (GITR or CD357), a TNFRSF member, is constitutively expressed on iNKT cells and is upregulated after activation. The effects of GITR signaling on iNKT cells is somewhat contested. A paper by Chen et al. shows that GITR has a co-inhibitory role in iNKT cell activation as demonstrated by decreased proliferation and cytokine production in WT mice compared with GITR-KO mice (38). However, GITR:GITRL interactions are necessary for Th1 and Th17 cytokine production by iNKT cells after stimulation by conventional DCs (25) and GITR stimulation using an agonistic monoclonal antibody enhances overall cytokine production by iNKT hybridomas *in vitro* (39). Further studies are needed to address these disparities found in the literature to determine the effects of GITR on iNKT cell activation.

OX40 (CD134), a TNFRSF member, is expressed on iNKT cells and interacts with OX40L on APCs but the outcome of this interaction is debated. In the pancreas, the OX40:OX40L interaction between iNKT cells and plasmacytoid DCs during LCMV infection, tested using neutralizing antibodies, induces IFN-α/β production by the DCs and dampens the adaptive immune response to avoid tissue damage (40). By contrast, stimulation of OX40 with an agonistic monoclonal antibody on liver-resident iNKT cells results in caspase-1-dependent pyroptosis and release of inflammatory cytokines that cause tissue injury (41). In a tumor model, iNKT cell expansion and IFN-γ production are enhanced by upregulation of OX40L on DCs (42). OX40 is stereotypically thought of as a stimulatory co-receptor, but its role in iNKT cell responses is unclear and may be tissue specific.

CD155, a member of the immunoglobulin superfamily, is expressed on iNKT cells and interacts with CD226, CD96, and TIGIT. CD155 blockade or knockout increases NKT1 cells and decreases both NKT2 and NKT17 cell generation during development in Balb/c and C57BL/6 mice (43). Its effect on iNKT cell activation and cytokine production has not been published.

There are three different T cell immunoglobulin mucin (TIM) receptors expressed by iNKT cells (TIM-1, 3, and 4), and they have differing effects on iNKT cell activation. TIM-1 engagement on iNKT cells by monoclonal antibodies suppresses Th1 responses but enhances Th2 responses in both *in vitro* and *in vivo* models (44). Conversely, TIM-1 engagement by phosphatidylserine—a marker of apoptosis—enhances iNKT cell activation, proliferation, and cytokine production (45). In a nonalcoholic fatty liver disease model, TIM-3 is shown to control liver-resident iNKT cell homeostasis with direct TIM-3 signaling inducing apoptosis and indirect signaling from IL-15, produced by TIM-3 stimulated Kupffer cells, promoting iNKT cell proliferation (46). TIM-3 is classically an inhibitory receptor and is upregulated on human iNKT cells in chronic viral infections (47). TIM-4 is expressed but does not have an effect on iNKT cell development or function (48). The effects of TIM-1 and TIM-3 need to be further assessed in iNKT cell biology.

B and T lymphocyte attenuator (BTLA), a member of the CD28 family that interacts with B7-H4, is an inhibitory co-receptor that is expressed on iNKT cells. Thus far, it has only been examined in ConA-induced hepatitis with both studies demonstrating that BTLA knockout increases iNKT cell cytokine production and exacerbates hepatitis (49, 50), indicating an inhibitory role of BTLA in iNKT cell function. Although these results align with the role of BTLA in conventional T cells, more research is needed to assess the role of BTLA in other immune models.

Lymphocyte activation gene (LAG)-3, a member of the immunoglobulin superfamily that interacts with MHC class II, is induced on iNKT cells after activation. It has an inhibitory affect with overexpression resulting in inhibition of proliferation due to cell cycle arrest (51). LAG-3 is upregulated on human iNKT cells in chronic viral infection and is associated with decreased cytokine production (52). These inhibitory effects are consistent with the effects of LAG-3 in conventional T cells.

Programmed death (PD)-1, a member of the CD28 family, is constitutively expressed on iNKT cells at low levels, rapidly upregulated after activation, and thought to play a role in iNKT cell anergy (53–55). Blockade of PD-1 signaling during iNKT cell activation enhances Th1 immunity (56). PD-1 interacts with both PD-L1 and PD-L2, with PD-L1 also being expressed on iNKT cells. Blockade of PD-L1 increases IFN-γ production in mice and humans whereas blockade of PD-L2 increases IL-4 and IL-13 production (57–59). In chronic viral infections and tumor models, human iNKT cells are dysfunctional—failing to proliferate or produce cytokines after activation—and have upregulated PD-1 (47, 60). Blockade of PD-1 signaling after iNKT cell activation and upregulation of PD-1 is debated with one paper showing ability to rescue anergy (55) and two others showing inability to rescue anergy (54, 61). iNKT cells require CD28 signaling to produce cytokines in the presence of PD-1:PD-L1 signaling (53). The role of the PD-1 pathway in iNKT cell function is distinctly inhibitory.

Due to the innate-like qualities of iNKT cells, it was at one time contested how co-receptors affected iNKT cell activation. The research summarized above demonstrates that iNKT cells are sensitive to both positive and negative signaling. Indeed, the context of these signals can have dramatic effects on the type of immune response generated. The next sections will explore the effects of co-stimulatory molecules on the ability of iNKT cells to mount an effective antitumor immune response.

## iNKT Cells in Cancer

The antitumor capabilities of iNKT cells were demonstrated soon after their discovery in 1987 (1). In fact, the 1993 discovery of their exogenous activating ligand, α-GalCer, was tested using a B16 melanoma model (62). Multiple papers noted the potent bioactivity of α-GalCer—including inducing lymphocyte proliferation, NK cell activation, fewer metastases, and prolonged lifespan of tumor-bearing mice. Increased survival was correlated with IL-2 and IFN-γ production, APC activation, NK cell activation, and tumor-specific CTL production (63–66). However, it was not until 1997 that the proliferative effects of α-GalCer were shown to be dependent on CD1d, Vβ8, and co-stimulatory molecules (CD40/CD40L, B7/CTLA-4/CD28) (10), linking iNKT cells to α-GalCer. A key piece of evidence was provided when surface plasmon resonance was used to prove that glycolipids such as α- and β-GalCer can bind both mouse CD1 and human CD1d (67). In 2000, the importance of iNKT cells in tumor immunosurveillance and initiation of the antitumor immune response was demonstrated using a carcinogen-induced tumor model in mice that had various lymphocyte subsets knocked out by gene targeting or depletion (68).

**Figure 2:**
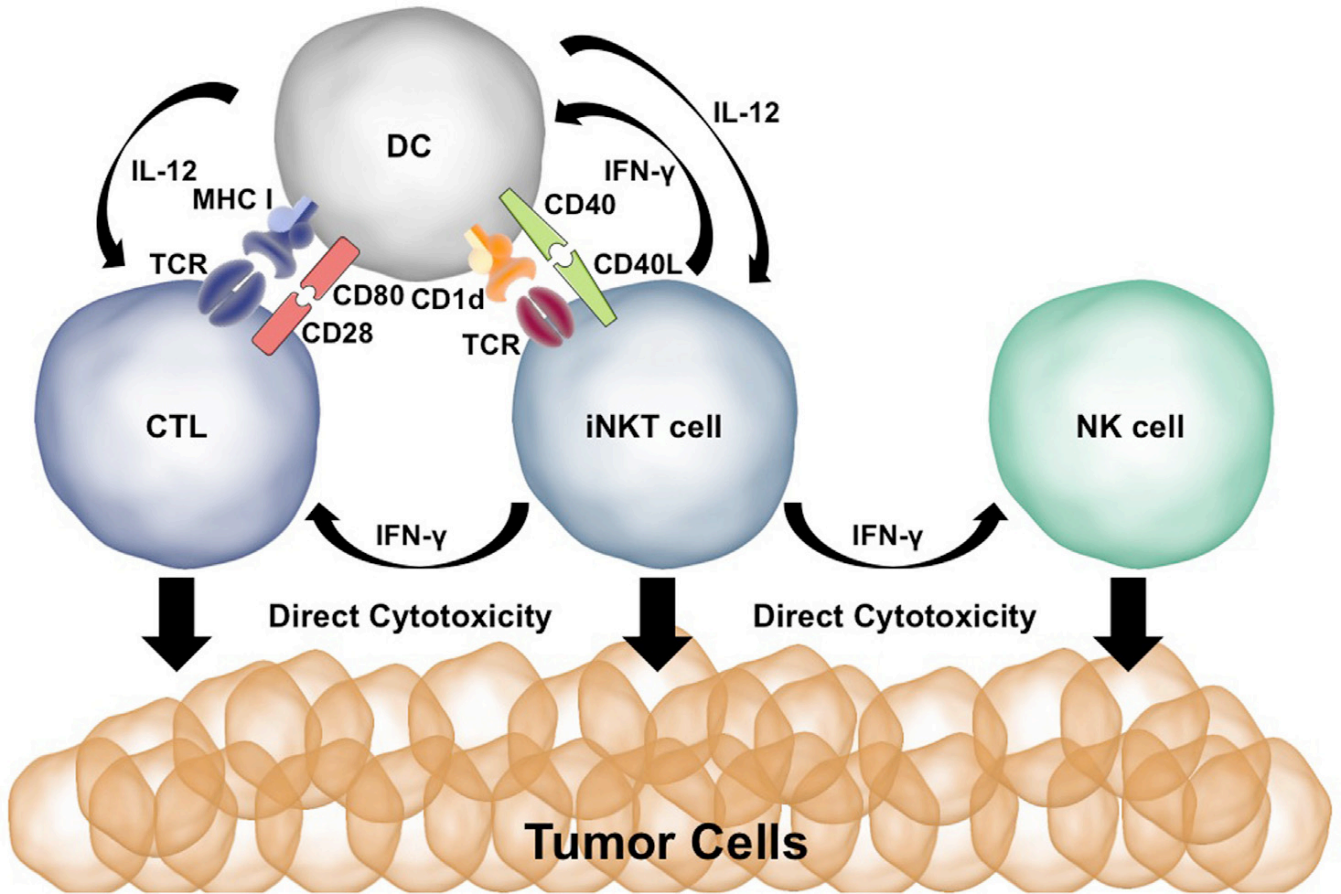
Co-stimulation plays a key role in the ability of invariant natural killer T (iNKT) cells to initiate antitumor immune responses. Presentation of α-GalCer to iNKT cells results in IFN-γ production. IFN-γ stimulates dendritic cells (DCs) to upregulate CD80/86 and activates natural killer (NK) cells. CD40L:CD40 interactions between the DC and iNKT cell activate the DC and result in IL-12 production. Cytotoxic T lymphocytes (CTLs) are activated by MHC:T cell receptor (TCR) interactions, CD80:CD28 co-stimulation, and IFN-γ and IL-12 signaling. iNKT cells, NK cells, and CTLs are able to directly kill tumor cells using perforin/granzyme and Fas:FasL.

Both CD28 and CD40 are needed to spur an effective antitumor immune response after α-GalCer injection (29, 30, 69). α-GalCer presentation to iNKT cells results in the production of IFN-γ and TNF-α. The CD40:CD40L interaction induces production of IL-12 by the DCs and upregulation of the IL-12Rα on iNKT cells. Activated iNKT cells can directly kill tumor cells *via* perforin/granzyme and Fas:FasL interactions (70, 71). Coadministration of α-GalCer and IL-12 works synergistically for iNKT activation, cytokine production, and cytotoxicity (29). IFN-γ release by iNKT cells activates NK cells to produce IFN-γ and directly kill tumor cells (72). IFN-γ and TNF-α upregulate CD80/86 on DCs whereas IL-12 induces a Th1/CTL immune response—promoting effective antitumor T cell immunity (73). Thus, malignant cells are killed directly by iNKT cells as well as indirectly *via* the activation of cytotoxic NK and CD8+ T cells. The potent effects of iNKT cells in antitumor immunity are summarized in Figure 2.

## iNKT Cells and Monoclonal Antibody Therapies

Progression of cancer to the stage of diagnosis indicates that the cancer has already undergone extensive immunoediting and has proceeded to the immune escape phase of immunosurveillance. In other words, cancers evolve to suppress and subvert the immune response (74). As reviewed by Joyce et al., the tumor microenvironment (TME) employs hypoxia, reactive nitrogen species, immunosuppressive chemokines and cytokines, dense extracellular matrix, and Th1-suppressive immune cells, such as regulatory T cells, myeloid-derived suppressor cells (MDSC), and tumor-associated macrophages, to suppress antitumor immunity (75). In fact, iNKT cells are frequently suppressed in cancer patients—displaying decreased cytokine production, cytolytic activity, and proliferation (76). Inhibitory co-receptor molecules are meant to stop aberrant immune responses such as autoimmunity (77). This system is termed a “checkpoint,” but tumors have hijacked expression of molecules such as PD-L1 to suppress and evade the antitumor immune response in humans and mice (78). A key branch of cancer immunotherapy research is on the use of monoclonal antibodies that block inhibitory co-receptor pathways (checkpoint inhibitors) and antibodies that engage co-stimulatory pathways to enhance antitumor immunity (79–85).

Two checkpoint inhibitor pathways have been extensively explored: CTLA-4 and PD-1. CTLA-4 is an inhibitory co-receptor that competes with CD28 for interaction with CD80/86. PD-1 and PD-L1 are both expressed on iNKT cells, but PD-1 engagement with PD-L1/L2 is inhibitory to iNKT cell function. Despite the popularity of CTLA-4 checkpoint inhibitors, research on the effects of α-CTLA-4 on iNKT cell activation is extremely limited. A set of papers from Pilones et al. came out examining the effects of iNKT cells on an immunotherapy regimen of radiation treatment and CTLA-4 blockade in a BALB/c breast cancer model (86, 87). This immunotherapy regimen is more successful in the absence of iNKT cells due to an increased influx of cross-presenting DCs in the tumor draining lymph node, but it is important to note that iNKT cell activation *via* α-GalCer administration is not included in the regimen. There has been slightly more research into PD-1/PD-L1 checkpoint inhibitors in tumor models. Checkpoint blockade of PD-1 or PD-L1, but not PD-L2, at the time of iNKT cell activation (by α-GalCer) increases cytokine production and cytotoxicity *in vitro* and *in vivo*, and decreases iNKT cell anergy, B16 melanoma tumor size, and metastatic lesions (54–56). It is still contested whether PD-1 blockade post-α-GalCer activation can rescue iNKT cells from anergy.

While checkpoint inhibitors have side effects such as autoimmunity, agonistic monoclonal antibodies against stimulatory co-receptors can cause rampant, destructive immune activation—making researchers more cautious with their use. Two such agonists, against members of the TNFRSF, have been explored in conjunction with iNKT cell immunotherapy: 4-1BB and GITR. In a mouse model of B cell lymphoma, treatment with α-GalCer-loaded, irradiated tumor cells and α-4-1BB increases overall survival and tumor-free survival dependent on IFN-γ and KLRG1+ CTLs (88). This immunotherapy also generates a memory immune response. Another group designed a therapy called NKTMab that includes α-4-1BB, α-DR5, and α-GalCer or α-C-GalCer (a modified version of α-GalCer known to skew the iNKT cell response to Th1). This combination immunotherapy causes effective rejection of 4T1 breast cancer tumors in Balb/c mice that is dependent on CD4+ T cells, CTLs, iNKT cells, and IFN-γ, and they found that α-C-GalCer was more effective in a wider range of concentrations (89). The role of GITR in iNKT cell mediated antitumor immune responses is not fully elucidated. In one paper using a C57Bl/6 T cell lymphoma model, iNKT cells in GITR-KO mice exhibit increased survival compared with WT mice (38). In a B16 melanoma model, mice treated with an agonistic mAb against GITR (DTA-1) exhibit increased survival that was dependent on NK1.1+ cells and T cells (90).

Checkpoint inhibitors have excelled in the clinic, but research into their effects on iNKT cells is lacking. Treatment regimens that combine iNKT cell activation and checkpoint blockade or agonistic antibody treatments hold promise for the future.

## Modified APCs

Antibody treatments can be harsh due to off-target effects. One method of co-stimulatory delivery is DC vaccines. DC vaccines have been researched and improved upon for decades, with the first cancer vaccine approved by the FDA in 2010. DCs provide co-stimulatory molecules in a more natural context—thus limiting off-target effects. Loading DCs with α-GalCer before vaccination enhances iNKT cell IFN-γ production and decreases tumor metastasis in B16 melanoma and Lewis lung carcinoma models (91, 92). In cancer patients, administration of α-GalCer-loaded DCs results in sustained iNKT cell expansion and enhanced antigen-specific T cell responses (93). Coadministration of irradiated tumor cells with α-GalCer or injection of α-GalCer-loaded, irradiated tumor cells enhances iNKT cell-mediated antitumor immune response *via* DC cross-presentation in plasmacytoma, lymphoma, and B16 melanoma models (94, 95). One vaccination strategy injects α-GalCer-loaded MDSCs—immunosuppressive immune cells created by the tumor—and demonstrates enhanced survival dependent on CTLs, NK cells, and iNKT cells. This enhanced immunity is due to increased positive co-stimulatory molecule (CD40, CD80/86) expression on the MDSC cell surface after iNKT cell interaction (96). Pretreatment of DCs with the Th1, pro-inflammatory cytokine TNF-α enhances positive co-stimulatory molecule expression such as CD80, CD86, 4-1BBL, and OX40L. OX40L expression drastically enhances antitumor immunity by enhancing iNKT cell activation, cytokine production, expansion, and stimulation of antitumor CTL responses (42). These papers demonstrate the impact of APC modification and how this influences iNKT cell mediated antitumor immunity.

## Chimeric Antigen Receptors (CARs) in iNKT Cells

In addition to checkpoint inhibition and modified APCs, another unique approach that takes advantage of the antitumor capabilities of iNKT cells involves the use of CARs. A CAR is an artificially engineered receptor containing an extracellular antigen recognition domain attached to an intracellular T cell activation domain. Traditionally, in cancer immunotherapy, CARs are placed in conventional T cells and contain an extracellular domain that recognizes a tumor antigen along with intracellular CD3ζ and co-stimulatory domains that provide the appropriate signals needed to fully activate the T cell against the tumor. First-generation CARs were composed of an extracellular single-chain variable fragment (scFv) and a CD3ζ, which meant they required endogenous co-stimulation for activation. Second and third generation CARs included one or two co-stimulatory signaling domains, respectively, in addition to the CD3ζ chain, which eliminated the need for endogenous co-stimulation (97).

However, there are several issues with using conventional T cells in CAR based cancer immunotherapy that may be overcome by expressing CARs in iNKT cells. One major complication is graft-versus-host disease (GVHD). Conventional TCRs are restricted to the polymorphic MHC (98), which can result in an allogenic anti-host response by donor T cells. By contrast, iNKT cells are restricted to the monomorphic CD1d molecule. Since CD1d is monomorphic, meaning it is conserved across individuals, iNKT cells can be adoptively transferred without concern for HLA matching (3, 4, 10). Another advantage iNKT cells have over conventional T cells is their ability to regulate off tumor effects. Several studies have reported that GVHD is exacerbated in CD1d−/− or Jα18−/− mice and that stimulation of iNKT cells can increase antileukemia responses while simultaneously mitigating the severity of GVHD (99, 100).

Human and mouse iNKT cells have the unique ability to secrete both Th1 and Th2 type cytokines, which may partly explain how they can simultaneously regulate GVHD and promote antitumor immunity (101, 102). Lee et al. showed that in humans, CD4+ iNKT cells were able to secrete the Th2 cytokines IL-4 and IL-13 whereas DN iNKT cells were able to secrete Th1 cytokines (103). They proposed that this may explain the ability of iNKT cells to facilitate both Th1 and Th2 type responses. A study later conducted by Tian et al. showed that stimulation with the combination of CD1d, CD86, 4-1BBL, and OX40L resulted in the greatest production of Th1 type cytokines by human CD19-specific CAR-iNKT cells containing a 4-1BB co-stimulatory domain (104). A future generation CAR containing the signaling domains of all these co-stimulatory molecules could be more effective at generating antitumor Th1 type responses.

Despite all the promising reasons to the use CAR-iNKT cells in cancer immunotherapy, there have been relatively few studies completed (105). However, the few studies that do exist have yielded promising results. In 2014, Heczey et al. generated a human anti-GD2 CAR-iNKT cell to target GD2+ neuroblastoma and found that these cells were able to localize to the tumor and initiate antitumor responses to neuroblastoma *in vivo* with no indication of the development of GVHD (106). Two years later, the same group generated anti-CD19 CAR-iNKT cells (104). CD19 is expressed on B cells and is being actively explored as a therapeutic target to treat various types of lymphoma derived from B cells. This study showed that anti-CD19 CAR-iNKT cells were able to selectively target CD19+ cells both *in vitro* and *in vivo*. In addition, they identified CD62L+ as a marker of the most effective CAR-iNKT cells due to greater proliferative potential and enhanced tumor reduction when compared with their CD62L− counterparts (104).

**Figure 3:**
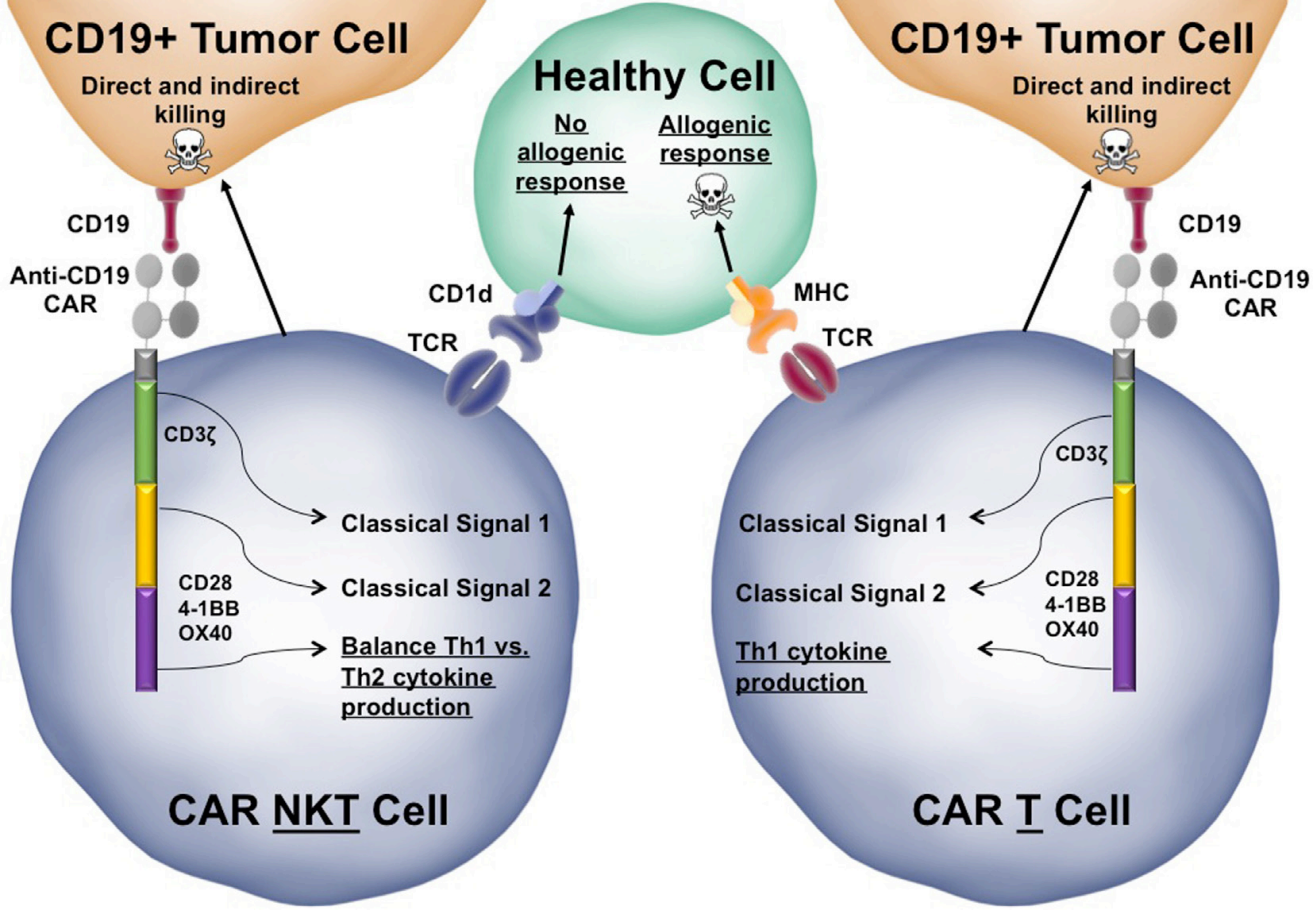
Functional advantages of chimeric antigen receptor (CAR)-invariant natural killer T (iNKT) cells over conventional CAR-T cells. The structure of a third-generation anti-CD19 CAR-iNKT cell is depicted interacting with both a target CD19+ tumor cells and a healthy bystander cell. Third generation CARs have three endodomains that can be modified to tune the response of the iNKT cell when activated. Similar to conventional iNKT cells, CAR-iNKT cells would be able to directly kill tumor cells using Fas:FasL interactions and secretion of perforin/granzyme. CAR-iNKT cells also secrete stimulatory cytokines, such as IFN-γ, that can license dendritic cells as well as indirectly activate cytotoxic T lymphocytes and natural killer cells to kill tumor cells (not depicted). The CAR-iNKT cells is juxtaposed to a similar CAR-T cell to highlight a few key differences between CARs in iNKT cells and CARs in conventional T cells (differences are underlined). Skull and crossbones indicate cell killing.

CAR-T cells are emerging as a powerful tool in the field of cancer immunotherapy. Given the current evidence suggesting that using iNKT cells may be able to overcome some of the problems associated with CAR-T cell therapy (highlighted in Figure 3), further study of CAR-iNKT cells, especially revolving around the use of various co-stimulatory domains to take advantage of their poly-functional cytokine secretion profiles, should prove rewarding. In addition, there have been other recent advances in the ability to isolate and expand human and mouse iNKT cells *ex vivo* for adoptive transfer that is beyond the scope of this review but will further facilitate the therapeutic use of these cells (36, 107, 108).

## Future Directions

The development of CARs has followed a predictable pattern of continuously trying to add more and more co-stimulatory domains to the receptor to enhance activation. To make these receptors as efficient as possible, it is worth exploring new possible co-stimulatory domains not traditionally included in CARs. A recent study conducted by Baglaenko et al. found a new role for Ly108 in iNKT cells (109). Ly108 has been previously established to play a role in iNKT cell development; however, this recent study found that peripheral trans-Ly108 interactions between APCs and iNKT cells enhanced the ability of iNKT cells to secrete cytokines and that loss of Ly108 expression resulted in defective iNKT cell homeostasis in mice. They went on to find that Ly108 activation in human iNKT cells led to increased secretion of the Th1 cytokines IFN-γ and TNF-α whereas levels of Th2 or regulatory cytokines, including IL-4 or IL-10, were unchanged. In addition to Ly108, there are several other innate-like co-stimulatory molecules such as TLR3, TLR9, and NKG2D that are known to be expressed on iNKT cells and be involved in immune surveillance. TLR3 and TLR9 agonists have been shown to enhance iNKT cell’s ability to mature DCs (110), whereas tumor cells are thought to shed NKG2D ligands in exosomes to block the receptor from recognizing the tumor cell (111). The unique signaling cascades and the involvement of adaptor proteins could complicate the use of these signaling domains in CARs. However, they enhance iNKT cell-mediated antitumor immunity, thus their potential may outweigh the costs.

We have primarily focused on co-stimulatory domains; however, it is also important to note the inhibitory domains and how they might be taken advantage of to enhance iNKT cell mediated antitumor immunity. Tumor cells will upregulate inhibitory molecules in response to inflammatory cytokines, which serve to inhibit any local antitumor immune response. One of the most well-known co-inhibitory molecules upregulated by many tumor types is PD-L1, which binds to PD-1 on activated immune cells to inhibit their function. Cherkassky et al. found that human CAR-T cells became exhausted due to tumor cell expression of PD-L1. They also found that CAR-T cell function could be rescued by anti-PD-1 therapy or by overexpression of a dominant negative PD-1 receptor. The dominant negative receptor consisted of the PD-1 extracellular ligand binding domain without any intracellular signaling domain (112).

Along the same lines as co-inhibitory molecules, blocking inhibitory cytokines commonly present in the TME, such as TGF-β, is being actively explored and has shown some promise (113). Terabe et al. found that CD11b+ or Gr1+ myeloid cells secreted TGF-β in a CD1d and IL-13 dependent manner and that removal of these cells prevented tumor recurrence, suggesting iNKT cells were actually playing a role in promoting an immunosuppressive environment (114). Directing the CAR against TGF-β could augment the ability of CAR-iNKT cells by simultaneously enhancing antitumor immunity while inhibiting immunosuppressive functions. Considering that there are already multiple known co-inhibitory receptors and cytokines, the possibility of including dominant negative receptors for each or even attaching CARs to co-inhibitory ligand binding domains provides new avenues of exploration to enhance iNKT cell-mediated antitumor immunity.

## Author Contributions

SS and ML equally contributed to the literature search and writing of this study. TW provided guidance for and editing of the manuscript. All the authors approved the final manuscript.

## Conflict of Interest Statement

TW is the founder and CEO of WebbCures, LLC and serves as a coeditor for this special issue. The other authors declare that the research was conducted in the absence of any commercial or financial relationships that could be construed as a potential conflict of interest.
